# Ski Tourism Shapes the Snow Microbiome on Ski Slopes in the Italian Central Alps

**DOI:** 10.1111/1758-2229.70195

**Published:** 2025-09-18

**Authors:** Andrea Nicolò Dell'Acqua, Daniel Scicchitano, Nicola Simoncini, Ilaria Mercanti, Daniela Leuzzi, Silvia Turroni, Luca Corlatti, Simone Rampelli, Martino Colonna, Cinzia Corinaldesi, Marco Candela, Giorgia Palladino

**Affiliations:** ^1^ Unit of Microbiome Science and Biotechnology, Department of Pharmacy and Biotechnology University of Bologna Bologna Italy; ^2^ Fano Marine Center The Inter‐Institute Center for Research on Marine Biodiversity, Resources and Biotechnologies Pesaro Urbino Italy; ^3^ Department of Life and Environmental Sciences Polytechnic University of Marche Ancona Italy; ^4^ Department of Sciences and Engineering of Materials, Environment and Urbanistics Polytechnic University of Marche Ancona Italy; ^5^ Stelvio National Park Bormio Italy; ^6^ University of Freiburg Freiburg im Breisgau Germany; ^7^ Sport Technology Lab Department of Civil, Chemical, Environmental and Materials Engineering Bologna Italy; ^8^ National Biodiversity Future Centre Palermo Italy

**Keywords:** anthropogenic impact, faecal contaminants, Italian Alps, ski tourism, snow microbiome

## Abstract

Winter sports exert significant anthropogenic pressures on the snow microbiome, affecting the entire alpine ecosystem. The massive usage of artificial snow, human occupation, and the release of xenobiotics like microplastics or ski wax components on ski tracks can profoundly alter snow microbial ecology. Here, we reconstructed the temporal dynamics of the snow microbiome at three sites in the Italian Alps: inside and outside a ski track at the impacted site of Santa Caterina Valfurva and near Cancano lake as an unimpacted control. Using epifluorescence microscopy, 16S rRNA amplicon sequencing, and inferred metagenomics, we found that the snow microbiome inside the track presented a higher load of prokaryotes and viruses. Notably, N_2_‐fixing microorganisms from cryospheric environments and host‐associated taxa, like *Terrisporobacter*, *Clostridium sensu stricto*, *Enterococcus*, and *Muribaculaceae*, and the opportunistic pathogen *Citrobacter* characterised the impacted site. These microorganisms could originate from the river water used to produce artificial snow during winter. Our findings highlight the complexity and multifunctionality of the snow microbiome, where microorganisms with different ecological propensities can coexist, and the detectable impact of ski tourism, which enriches host‐associated and xenobiotic‐degrading microorganisms. This underscores the need for systematic monitoring and protection of the snow microbiome in the Alpine environment from anthropogenic threats.

## Graphical abstract

Ski tourism alters the snow microbiome by increasing microbial load and introducing human‐ and host‐associated taxa. These shifts may impact alpine ecosystem functions and highlight the need for systematic microbiome monitoring in mountain environments.

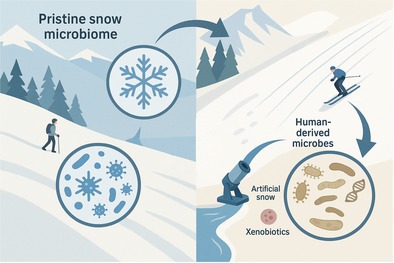

## 1 Introduction

The Alps are the world's most popular ski destination, accounting for 43% of the global ski tourism with an annual turnover of 33 billion euros in 2020 (Vanat 2020). This results in massive anthropogenic pressure on alpine ecosystems (Grassl 2002; Vigl et al. 2021), threatening local biodiversity and compromising the provision of key ecosystem services needed for environmental, animal, and human health. This is particularly true for the alpine environmental microbiomes (Wang et al. 2022) as a life support system for the alpine trophic chain, which is threatened by global changes (Cavicchioli et al. 2019).

In particular, the alpine snow microbiome is a highly dynamic ecosystem (Bourquin et al. 2022), in which actively metabolising, cold‐adapted and UV‐resistant microorganisms, such as photoautotrophs and chemosynthetic lithotrophs, coexist with heterotrophic generalists not necessarily adapted to the cold environment and frequently found in temperate freshwaters and soils (Zhu et al. 2020; Fillinger et al. 2021). The establishment of this complex microbial ecosystem in pristine snow primarily involves seeding of atmospheric microorganisms by deposition (Malard et al. 2023). The phyllospheric microbiomes from the surrounding forests are another important source of microorganisms (Sanchez‐Cid et al. 2022). Finally, host‐associated microbiomes resulting from animal faecal contamination can be another relevant component of the snow microbial community (Staley et al. 2017; Kalinowska et al. 2021; Niu et al. 2023).

The psychrophilic microorganisms active in snow are involved in a range of microbial processes, such as carbon and nitrogen fixation and the decomposition of organic matter deposited from atmospheric dust and forest litter, playing an important role in biogeochemical processes (Zhu et al. 2020; Bourquin et al. 2022). At melting, all snow microorganisms inevitably encounter soil microbial communities (Malard et al. 2023). Indeed, the snowpack is an important source of new potential colonisers for the soil microbiome, providing up to 10^4^ microbial cells per mL of surface snow at different latitudes (Zhang et al. 2010; Cameron et al. 2015; Fillinger et al. 2021). A similar abundance was found for viral particles in the alpine snowpack (Fillinger et al. 2021). Snow melting also results in a peak of nutrients and solutes in the soil, providing an opportunity for snow microorganisms to get established in the new habitat and become an integral part of the soil microbial community. Given these dynamics, it is clear that the ecological role of the snow microbiome extends beyond the cryosphere, being essential for biochemical processes and nutrient cycling in soil and freshwater ecosystems, including alpine ecosystems (Fillinger et al. 2021).

Winter tourism and winter sports, such as skiing and snowboarding, might shape the compositional layout of the snow microbiome in ski resorts (Sanchez‐Cid et al. 2022). In particular, anthropogenic impacts on the snow microbiome may be the result of several factors. For example, the massive use of artificial snow for slope preparation, now exacerbated by climate change (Carrer et al. 2023), may lead to profound changes in the snow microenvironment, with downstream effects on the resident microbial community. Compared to natural snow, artificial snow originates from close by river water and has a higher density, a lower O_2_ concentration and an increased abundance of several chemical elements, such as Mg^2+^, SO_4_^2−^, Ca^2+^, K^+^, Cl^−^ and Na^+^, potentially altering the competitive balance between microbiome components by favouring faster‐growing species in rich environments (Kammer 2002; Baloh et al. 2019). Moreover, human presence on ski runs might favour the dispersal and deposition of human‐associated bacteria in the snow environment, possibly including pathogens that can persist in the snow and be transported to local soils and water bodies when the snow melts (Kammer 2002). Finally, the release of microplastics and other xenobiotics, such as ski wax components, on ski runs might represent a selective force for degrading microorganisms (Ásmundsdóttir and Schulz 2020; Rüthi et al. 2023). Indeed, microplastics have been detected in snow, particularly in anthropised environments (Bergmann et al. 2019; Parolini et al. 2021; Ohno and Iizuka 2023). Such materials can be released from winter sports equipment, outdoor winter clothing used by skiers and grooming and snow‐making machines, which are composed entirely or in part of polymers (Colonna et al. 2013; Dasari et al. 2016). In particular, polyethylene is the main component of ski‐bases, ABS (acrylonitrile–butadiene–styrene) is the main component of ski side walls, polyurethanes are the most commonly used materials for ski boots and grooming machine belts, while polyamides, polyesters and polyurethanes (elastane) are the most commonly used fibres for ski clothing.

Although this scenario suggests the need to monitor the impact of winter tourism on the snow microbiome, studies in this direction are still fragmentary and current knowledge of the impact of winter tourism on the Alpine environmental microbiomes is still lacking.

In order to provide some insight into this issue, here we explored the temporal variation of the snow microbiome inside and outside a ski track at the Santa Caterina Valfurva ski area, as an anthropogenically impacted site, and at a site near Cancano lake, characterised by low anthropogenic impact (used as a control). In particular, we combined a next‐generation sequencing (NGS) approach of the 16S rRNA gene to characterise the microbiome composition with a microscopy‐based approach to determine the abundance of microbes, including viruses. Our findings provide new information on the snow microbiome changes on ski tracks for predicting some possible ecological implications of winter tourism.

## 2 Materials and Methods

### 2.1 Site Description, Sampling, and Environmental Data

Snow samples were collected during the ski season from December 2021 to April 2022. They were collected monthly at three different sites within the Stelvio National Park (Central Italian Alps): inside (IN site) and 10 m outside (OUT site) a ski slope at Santa Caterina Valfurva (46°24′15″ N 10°28′589″E), and at the unaffected reference site near Cancano Lake (REF site, 46°30′59″N 10°18′49″E). The Santa Caterina Valfurva ski resort is located between the town of Santa Caterina at 1738 m a.s.l. (above sea level) and Monte Sobretta at 2880 m a.s.l. The resort has about 14 ski runs, for a total of about 35 km of slopes. During the 2021–22 winter season, about 145,000 skiers visited the area. The sampling sites were located at 2109 m a.s.l., close to a ski lift. The site is exposed to the north, with a slope of about 20°; it lies on metamorphic rocks and the surrounding vegetation is characterised by the presence of hay meadows and coniferous forest dominated by Norway spruce *Picea abies* . During the winter season, the ski slopes (including the one where the sampling site was located) can be artificially snowed with water from a nearby stream (Torrente Frigidolfo) that originates at the Gavia Pass, an area grazed by cattle during the summer season. The unaffected reference site of Cancano was located at approximately the same altitude as the sampling site of Santa Caterina (1936 m a.s.l.). The area is characterised by a northern exposure, with an inclination of less than 10°; the site is located on calcareous soil and the surrounding vegetation is characterised by the presence of alpine meadows and coniferous forest of mountain pine *Pinus mugo* . Unlike the Santa Caterina sampling sites, the reference site area is closed to vehicles during the winter season and the presence of people is limited to park rangers and dam keepers; however, the sampling site was not visited by people during the sampling period. All sampling sites are frequented by the main vertebrate species of the park, such as red deer *Cervus elaphus* , roe deer *Capreolus capreolus* , chamois *Rupicapra rupicapra* , and hare *Lepus* spp. At the time of sampling, the three sites experienced similar meteorological conditions (Figure S1). The collection of surface snow (approximately the first 10 cm depth) was performed with a previously sterilised small shovel. For microbiological characterisation, during sampling in December 2021, we collected four samples at the pristine site (REF), two samples at the ski track site (IN) and two samples outside the ski track (OUT), all in 1‐L sterile and sealed plastic bags. For sampling from January to April, we collected two samples at the REF site, one sample at the IN site, and one sample at the OUT site placed into 2 L sterile and sealed plastic bags. For prokaryotic and viral abundances, an additional 2 L of surface snow was collected from each site for further microscopic analysis. All samples were immediately transported to the Stelvio National Park facilities, where they were stored at −20°C until shipment. Finally, samples for microbiological analysis were shipped iced to the University of Bologna, while samples for bacterial and viral load were shipped iced to the Marche Polytechnic University. Upon arrival, the samples were stored at −20°C until further processing. Snowfall on the ground (cm) from November 2021 to May 2022 was recorded through meteorological stations close to the sampling sites (Figure S1).

### 2.2 Epifluorescence Microscopy to Determine Microbial Abundance

Prokaryotic and viral abundances in the snow samples were determined according to Noble and Fuhrman (1998) with minor modifications and in accordance with previously established procedures (Dell'Anno et al. 2009; Patel et al. 2007; Corinaldesi et al. 2019). Samples were melted and the resulting water was homogenised in sterile Whirl‐Pak bags. To exclude the presence of other particles (e.g., microplastics, metals, dust), subsamples (150 mL) from each sample were filtered through a previously sterilised 20‐μm mesh (Sefar, Thal, Swizerland). From each filtered sample, three replicates (5 mL each) were used for analysis of prokaryotic and viral abundances. DNAse I from bovine pancreas (final concentration, 2 U/mL) was added to subsamples, which were then incubated for 15 min at room temperature (20°C). Each subsample was then filtered onto 0.02‐μm Anodisc filters (Whatman), stained with 100 μL SYBR Gold I (final concentration 2×; SIGMA‐Aldrich, St. Louis, MO, Stati Uniti) and incubated for 20 min in the dark. After staining, the filter was mounted on a microscope slide with the addition of 20 μL of antifade solution (1:1 phosphate buffer:glycerol, containing 0.5% ascorbic acid, pH 7.8). Prokaryotic and viral counts were performed under epifluorescence microscopy (Zeiss Axioplan; magnification, 1000×) by examining at least 20 fields per slide. Analysis of variance (ANOVA) was used to investigate differences in prokaryotic and viral abundances among stations (factor type: REF, IN, OUT) and between sampling months (factor month: Jan, Mar). Whenever factors were identified as significant, an HSD‐Tukey post hoc test was performed. All statistical analyses were performed using PAST software (v.4.14).

### 2.3 Microbial DNA Extraction and 16S rRNA Amplicon Sequencing

The collected snow was melted at 4°C and then processed by vacuum filtration under sterile conditions using 0.22‐μm pore size MF‐Millipore (Darmstadt, Germany) membrane filters. Microbial DNA was extracted using the DNeasy PowerWater Kit (Qiagen, Hilden, Germany) following the manufacturer's instructions. A negative control for the extraction process was carried out together with the samples. The extracted DNA was quantified using a NanoDrop ND‐1000 spectrophotometer (NanoDrop Technologies, Wilmington, DE, USA) and stored at −20°C until further processing.

Library preparation was performed according to the Illumina 16S Sequencing Library Preparation protocol (Illumina, San Diego, CA, USA). The V3–V4 hypervariable region of the 16S rRNA gene was amplified by PCR in a 50‐μL final volume, containing 25 ng of microbial DNA, 2× KAPA HiFi HotStart ReadyMix (Roche, Basel, Switzerland), and 200 nmol/L forward 314 and reverse 785 primers carrying Illumina overhang adapter sequences (Klindworth et al. 2013). The PCR thermocycle consisted of 3 min at 95°C, followed by 30 cycles of 30 s at 95°C, 30 s at 55°C and 30 s at 72°C, and a final elongation step at 72°C for 5 min (Scicchitano et al. 2022). A negative PCR control was carried out together with the samples. Amplified products were purified using Agencourt AMPure XP magnetic beads (Beckman Coulter, Brea, CA, USA). Indexed libraries were prepared by limited‐cycle PCR using Nextera technology (Illumina) and purified again as described above. The libraries were then quantified using a Qubit 3.0 fluorimeter (Invitrogen, Waltham, MA, USA), normalised to 4 nM, and pooled. Finally, the library pool was denatured with 0.2 N NaOH and diluted to 4.5 pM with a 20% PhiX control. Sequencing was performed on an Illumina MiSeq platform using a 2 × 250‐bp paired‐end protocol, according to the manufacturer's instructions (Illumina).

### 2.4 Bioinformatics and Biostatistics

Raw sequences were processed using a combination of the PANDAseq (Masella et al. 2012) and QIIME 2 pipelines (Bokulich et al. 2018). The ‘fastq filter’ function of the Usearch11 (Edgar 2010) algorithm was applied to retain high‐quality sequences. Specifically, based on the probabilities of the phred Q score, sequences with an expected error per base *E* = 0.03 (i.e., three expected errors per 100 bases) or higher were discarded. High‐quality sequences were then clustered into amplicon sequence variants (ASVs) using DADA2 (Callahan et al. 2016). After the DADA2 step, potential environmental or laboratory contamination was removed by identifying and excluding ASVs that were also detected in negative controls (DNA extraction negative control and PCR negative control), which were processed alongside the original samples. A normalisation step based on alpha rarefaction curve analysis was performed to ensure comparability across samples, resulting in the exclusion of two samples (February REF site and December IN site). Taxonomic assignment was performed using a hybrid method combining the VSEARCH (Rognes et al. 2016) algorithm and the q2‐feature‐classifier plugin (Bokulich et al. 2018) trained on the SILVA database (2020, v138.1) (Quast et al. 2012). All sequences assigned to eukaryotes and unassigned sequences (not assigned to prokaryotes) were discarded. Alpha diversity was calculated using Shannon diversity, Simpson and Inverted Simpson indices, the number of observed taxa, and Faith's phylogenetic diversity at the ASV level. Beta diversity was calculated using weighted and unweighted UniFrac distances at the ASV level and Bray–Curtis distances at different taxonomic levels.

All statistical analyses were performed using R software (R Core Team; http://www.r‐project.org), v.4.3.2, with the packages ‘vegan’ (https://cran.r‐project.org/web/packages/vegan/index.html), ‘ggplot2’ (Wickham and Wickham 2016), ‘reshape2’ (Wickham 2007), ‘RColorBrewer’ v.1.1–3 (https://CRAN.R‐project.org/package=RColorBrewer) and ‘gplots’ v. 3.1.3.1 (https://CRAN.R‐project.org/package=gplots). Data separation in principal coordinates analysis (PCoA) was tested using a permutation test with pseudo‐*F* ratio (function ‘adonis’ in vegan, 999 permutations). The Wilcoxon rank‐sum test was used to assess differences in alpha diversity. *P*‐values were corrected for multiple testing using the Benjamini–Hochberg method, with a false discovery rate ≤ 0.05 considered statistically significant.

Linear discriminant analysis (LDA) effect size (LEfSe) (Segata et al. 2011), aimed at identifying discriminant taxa between sites, was performed on ASV relative abundance tables, retaining only taxa with an LDA score threshold of ±2 (on a log_10_ scale) and a *p* ≤ 0.05. We then used BLASTn (last accessed July 2024) (Altschul et al. 1990) to identify bacterial species corresponding to the ASV sequences of the discriminant genera identified by LEfSe and potentially host associated.

PICRUSt2 (Douglas et al. 2020) with default parameters was used to predict metagenome functions based on the ASVs identified in our dataset. The output file with the predicted KO (KEGG orthology) copy number per ASV was used to construct the heatmap representing the metagenome functions, grouped by pathway, for the discriminant ASVs identified by LEfSe. ASVs were then clustered at 97% similarity to reconstruct shared ASV networks, which were constructed on the data provided by the *make_otu_network* script of the QIIME pipeline. Shared ASVs were defined as sequences present in at least three out of five sampling timepoints for each site. Plots were generated using Cytoscape software (http://www.cytoscape.org/) and the Compound Spring Embedder (CoSE) as the layout for the ASVs (Ruiz et al. 2023).

## 3 Results

### 3.1 Prokaryotic and Viral Abundances in Snow Samples at Impacted and Unimpacted Sites

Prokaryotic and viral abundances at the impacted sites inside (IN), 10 m outside (OUT) the ski track at Santa Caterina Valfurva, and at the control site (REF) in January and March are reported in Table 1 (see also Table S1 and Figure S2). Snow on the ground (cm) from November 2021 to May 2022 for the sites of Santa Caterina Valfurva and Cancano is reported in Figure S1. In January, prokaryotic abundances at the IN and OUT sites were not significantly different (average, 5.19 × 10^4^ cells/mL), but higher than at the REF site (9.64 ± 1.16 × 10^3^ cells/mL) (two‐way ANOVA test, *p* ≤ 0.05 for both). In March, the highest prokaryotic abundances (8.12 ± 0.49 × 10^4^ cells/mL) were found at the IN site (two‐way ANOVA test, *p*‐ ≤ 0.05 for the comparison against both OUT and REF sites). Similarly, viral abundances were higher at the IN site in both January (2.79 ± 0.23 × 10^5^ viruses/mL) and March (4.38 ± 0.21 × 10^5^ viruses/mL) compared to the other sites (two‐way ANOVA test, *p* ≤ 0.05 for the comparison against both OUT and REF sites in both January and March). The lowest values were observed at the REF site (January: 7.77 ± 0.46 × 10^4^ viruses/mL; March: 1.49 ± 0.10 × 10^5^ viruses/mL) (two‐way ANOVA test, *p* ≤ 0.05 for the comparison against both IN and OUT sites in both January and March). All prokaryotic and viral abundances were significantly higher in March (two‐way ANOVA test, *p* ≤ 0.05 for both prokaryotic and viral abundances).

**TABLE 1:** Prokaryotic and viral abundances.

	Prokaryotic abundance (cells/mL)			Viral abundance (viruses/mL)		
	REF	IN	OUT	REF	IN	OUT
January (avg ± SD)	9.64E+03	5.78E+04	4.60E+04	7.77E+04	2.79E+05	1.54E+05
	1.16E+03	7.38E+03	7.05E+03	4.61E+03	2.29E+04	1.47E+04
March (avg ± SD)	2.83E+04	8.12E+04	5.92E+04	1.49E+05	4.38E+05	2.91E+05
	2.90E+03	4.91E+03	4.66E+03	1.01E+04	2.12E+04	2.24E+04

*Note:* Mean (avg) and standard deviation (SD) values are reported for January and March 2022.

Abbreviations: IN, on‐track site; OUT, off‐track site; REF, reference site.

### 3.2 Microbial Ecology of Snow at Impacted and Unimpacted Sites

The structure and temporal dynamics of the snow microbiome at the IN, OUT and REF sites were reconstructed from December 2021 to March 2022 using 16S rRNA gene NGS. Snow samples were collected monthly from December 2021 to March 2022, both at the impacted sites in the Santa Caterina Valfurva ski area and at the unimpacted site of Cancano lake. A total of 30,926 reads from 24 samples (mean ± SD, 1288 ± 1914) and 618 ASVs were obtained.

**FIGURE 1:**
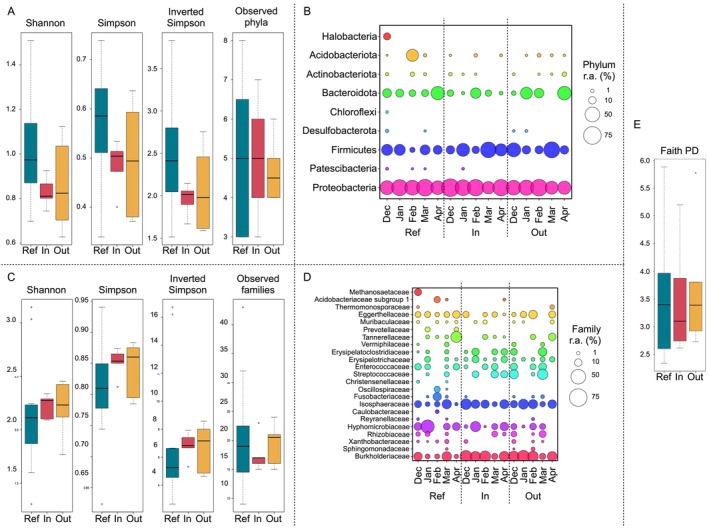
Compositional variation of the snow microbiome across sampling sites and timepoints. Boxplots showing the distribution of alpha diversity at the phylum (A) and family (C) level, based on the Shannon, Simpson and Inverted Simpson indices and the number of observed taxa, of the snow microbiome at the reference site (‘Ref’) and at the two impacted sites (‘In’ and ‘Out’ of the ski track). (E) Faith's phylogenetic diversity metric at the ASV level is also reported (Faith PD). No significant differences were found for any of the metrics (Kruskal–Wallis test, *p* > 0.05). Metacommunity bubble plot of the compositional structure of the snow microbiome at the phylum (B) and family (D) level at the three sampling sites in the different sampling months (from December 2021 to April 2022). The size of the bubbles represents the percentage of relative abundance according to the legend on the right.

The snow microbiomes at the IN and OUT sites showed an overall lower phylum‐level diversity than at the REF site, particularly according to the Shannon and Simpson diversity metrics (Figure 1A). Specifically, the IN and OUT microbiomes were dominated by Proteobacteria, Firmicutes and Bacteroidota, with Actinobacteriota and Acidobacteriota as subdominant phyla, while the REF microbiome also showed a relevant relative abundance of Halobacteriota, but only in December (Figure 1B). Interestingly, when considering quantitative diversity metrics at lower taxonomic levels (i.e., family), such as Shannon and Simpson, we observed the opposite trend, with the IN and OUT microbiomes being more diverse than the REF microbiome (Figure 1C). To evaluate whether these differences also reflected broader evolutionary diversity, we calculated Faith's phylogenetic diversity, a metric incorporating phylogenetic relationships among ASVs, regardless of their abundance. This analysis did not reveal significant differences across sites (Figure 1E), suggesting that the observed community differences may primarily stem from variations in the distribution of dominant components, rather than overall phylogenetic diversity.

The family‐level compositional structure of the snow microbiomes at each site across timepoints is shown in Figure 1D.

**FIGURE 2:**
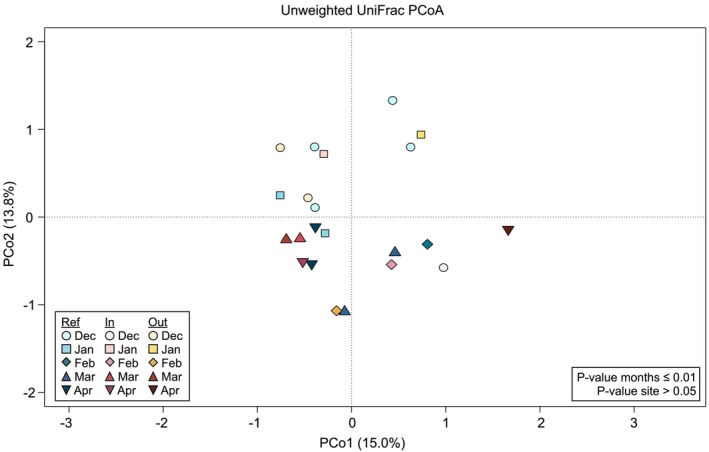
Beta diversity of the snow microbiome across sampling sites and timepoints. Principal coordinates analysis (PCoA) based on unweighted UniFrac distances between the microbial profiles of snow collected at the reference site (‘Ref’) and at the two impacted sites (‘In’ and ‘Out’ of the ski track) in the different sampling months (from December 2021 to April 2022). The first and second principal components (PCo1 and PCo2) are plotted, and the percentage of variance in the dataset explained by each axis is shown. Permutation test with pseudo‐*F* ratio, *p* ≤ 0.01 for comparison between sampling months, *p* > 0.05 for comparison between sampling sites.

The unweighted UniFrac PCoA plot at the ASV level showed significant segregation of samples by timepoint (permutation test with pseudo‐*F* ratio, *p* ≤ 0.01 for comparison between sampling months, *p* > 0.05 for comparison between sampling sites) (Figure 2), suggesting that variation in snow microbiomes was primarily driven by sampling month. However, no segregation was found in the Bray–Curtis‐based PCoA plot at the family level (permutation test with pseudo‐*F* ratio, *p* > 0.05 for all comparisons) (Figure S3), suggesting that the observed temporal variation of the snow microbiome at the different sites was likely due to the rearrangement of different ASVs belonging to the same families.

**FIGURE 3:**
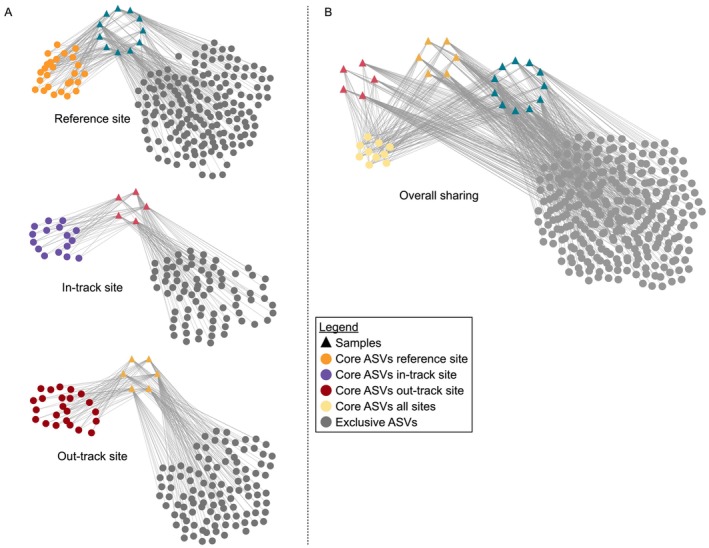
Networks of shared ASVs between sampling sites and timepoints. (A) ASVs clustered at 97% similarity shared between the different timepoints at the reference site (top), the in‐track impacted site (middle) and the out‐track impacted site (bottom). (B) ASVs clustered at 97% similarity shared among all sampling sites encompassing the entire sampling period (from December 2021 to April 2022). Dot and triangle size is fixed; colour legend of samples and shared or exclusive ASVs is shown at bottom right.

To further explore the temporal dynamics, we constructed network plots of the ASVs shared between the different timepoints at the different sites (Figure 3A). For each site, the taxonomy of shared ASVs is provided in Table S2. Although the vast majority of ASVs were variable over time and between sites, 10 ASVs were shared across all samples, potentially representing core snow microbiome components (Figure 3B). Overall, taxa corresponding to core ASVs accounted for 48.2% ± 12.5% (relative abundance, mean ± SD) of the total snow microbiome. Interestingly, these putative core taxa included known environmental and plant rhizosphere‐associated bacteria, which are frequently detected in the cryosphere environment (namely, *Sphingomonas*, *Pedobacter*, *Delftia*, and *Stenotrophomonas*) (Balkwill et al. 2006; Ryan et al. 2009; Antony et al. 2016; Bhat et al. 2022; Soto et al. 2022; Margesin et al. 2003), and host‐associated taxa, such as *Escherichia–Shigella*, *Klebsiella*, *Enterococcus*, and *Terrisporobacter* (Dancer et al. 1997; Lebreton et al. 2014; Niu et al. 2023). Other host‐associated taxa (*Muribaculaceae*, *Prevotellaceae*, *Clostridium sensu stricto 1* and *13* and *Citrobacter*) (Dancer et al. 1997; Lebreton et al. 2014; Niu et al. 2023) were identified as characteristic of the impacted site (Table 2). It should be noted that the relative abundance of host‐associated taxa fluctuated over time and showed different behaviour between sites. For example, the highest relative abundance of *Escherichia–Shigella* and *Klebsiella* was detected in January at the REF site (14.97% and 13.02%, respectively), that of *Enterococcus* in March at the IN site (32.32%), and that of *Terrisporobacter* in March at the OUT site (23.05%).

**TABLE 2:** Snow microbiome members belonging to host‐associated taxa.

Bacterial taxa	December			January			February			March			April		
	Reference	In‐track	Out‐track	Reference	In‐track	Out‐track	Reference	In‐track	Out‐track	Reference	In‐track	Out‐track	Reference	In‐track	Out‐track
Muribaculaceae	0.67	0.00	0.45	0.00	0.90	0.30	0.90	2.10	0.00	0.30	0.00	0.00	0.90	0.00	1.80
Prevotellaceae	0.00	0.00	0.00	3.89	0.00	0.00	0.00	0.00	0.00	0.00	0.00	0.00	2.69	0.00	0.00
Escherichia–Shigella	10.48	1.50	1.20	14.97	3.59	0.90	0.00	0.30	4.79	6.44	0.00	0.00	0.15	2.40	0.00
Klebsiella	0.00	0.00	4.94	13.02	7.19	0.00	0.00	0.00	1.20	0.60	6.29	6.29	10.93	4.79	0.00
Enterococcus	1.35	7.78	6.29	8.83	7.78	0.00	0.00	0.00	3.29	4.64	32.34	5.39	7.04	9.28	0.30
Terrisporobacter	0.00	0.00	16.32	0.45	8.08	0.00	0.00	0.00	0.60	3.29	10.48	23.05	0.00	8.68	0.00
Clostridium_sensu_stricto_1	0.22	1.20	7.19	1.50	10.48	0.00	0.00	0.00	0.00	2.40	5.69	11.98	0.00	7.49	0.00
Citrobacter	0.00	0.00	0.00	0.00	2.10	0.00	0.00	0.00	0.00	0.90	0.00	0.00	0.00	1.20	0.00
Clostridium_sensu_stricto_13	0.00	0.00	1.20	0.00	0.00	0.00	0.00	0.00	0.00	0.00	0.00	2.69	0.00	0.00	0.00

*Note:* The relative abundance (%) of host‐associated taxa is represented for each sampling time at the three sites. Core microbial components are *Escherichia–Shigella*, *Enterococcus*, *Terrisporobacter* and *Klebsiella*, whereas taxa characterising impacted sites are *Muribaculaceae*, *Prevotellaceae*, *Clostridium sensu stricto 1* and *13* and *Citrobacter*.

### 3.3 Anthropogenic Fingerprint of the Snow Microbiome on the Ski Track

**FIGURE 4:**
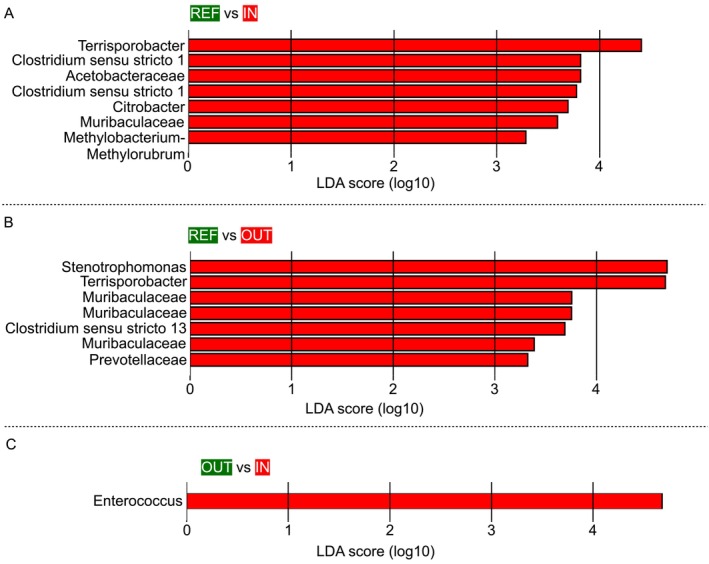
Discriminating ASVs between the different sampling sites. (A) Comparison between the reference site (REF) and the in‐track impacted site (IN). (B) Comparison between the reference site (REF) and the out‐track impacted site (OUT). (C) Comparison between the in‐track impacted site (IN) and the out‐track impacted site (OUT). The logarithmic threshold for discriminating features was set to 2.0 with *p* < 0.05. Plots were obtained by linear discriminant analysis (LDA) effect size (LEfSe) analysis performed on ASV relative abundance tables. BLASTn was then used to identify bacterial species corresponding to the ASV sequences of the discriminant genera identified by LEfSe and potentially host‐associated.

To identify the snow microbiome features that distinguish the different sites, a LEfSe analysis was performed. The results showed that, compared to the REF site, the IN site's snow microbiome was characterised by the presence of ASVs assigned to *Terrisporibacter*, *Clostridium sensu stricto 1*, *Acetobacteraceae*, *Citrobacter*, *Muribaculaceae* and *Methylobacterium–Methylorubrum* (Figure 4A). In contrast, the OUT site was distinguished by ASVs belonging to *Stenotrophomonas*, *Terrisporobacter*, *Muribaculaceae*, *Clostridium sensu stricto 13* and *Prevotellaceae* (Figure 4B). Notably, no ASVs were found to specifically characterise the unimpacted (REF) site in any comparison. Finally, when directly comparing the IN and OUT sites, the IN site was specifically characterised by ASVs assigned to *Enterococcus* (Figure 4C).

To explore possible functional implications arising from the snow microbiome compositional specificities at the impacted site, we inferred the metagenome of the IN and OUT discriminating ASVs by PICRUSt2 (Figure S4). Interestingly, the ASVs characterising the IN and OUT sites were endowed with functions involved in bacterial infection processes and degradation of xenobiotics, such as benzoate, bisphenol, styrene, and caprolactam, possibly contaminating the ski tracks.

## 4 Discussion

In the present work, we explored the impact of ski tourism on the snow microbiome in the Italian Alps. To this end, the temporal dynamics of snow microbial communities were reconstructed over 5 months during the winter tourist season at three sites, namely inside and outside a ski track at the anthropogenically impacted site of Santa Caterina Valfurva and at a site near Cancano lake, characterised by low anthropogenic impact (used as a control).

First, we assessed the microbial abundances (in terms of prokaryotes and viruses) in the snow samples by means of epifluorescence microscopy. Overall, our results are consistent with previous findings (Fillinger et al. 2021) and show an increasing pattern over time. In particular, the highest levels of both prokaryotes and viruses were found in March at the site most impacted by ski sports (inside the track), suggesting a microbial enrichment of the surface snow towards the end of the ski season.

We then used NGS to explore changes in snow microbiome structure over time at the different sites. The temporal variation in the snow microbiome compositional structure was found to be greater than the variation between sites, but mainly involved changes at low taxonomic ranks, resulting from the rearrangement of ASVs belonging to the same families. Nevertheless, we were able to identify a core snow microbiome, consisting of 10 ASVs that were shared between the different timepoints and sites. The core ASVs belonged to known cryospheric microbes, such as *Sphingomonas*, *Pedobacter*, *Delftia*, and *Stenotrophomonas* (Balkwill et al. 2006; Ryan et al. 2009; Antony et al. 2016; Bhat et al. 2022; Soto et al. 2022; Margesin et al. 2003), and host‐associated taxa, such as *Escherichia–Shigella*, *Klebsiella*, *Enterococcus*, and *Terrisporobacter* (Dancer et al. 1997; Lebreton et al. 2014; Niu et al. 2023). More specifically, *Sphingomonas* and *Pedobacter* are cold‐adapted microorganisms that are highly abundant in snowpack and can utilise a wide range of naturally occurring organic compounds (White et al. 1996; Balkwill et al. 2006; Antony et al. 2016; Soto et al. 2022; Margesin et al. 2003). Differently, *Delftia* and *Stenotrophomonas* are non‐psychrophilic ubiquitous environmental bacteria with important roles in N fixation. In association with plants, they can also act as plant growth‐promoting bacteria (Ryan et al. 2009; Bhat et al. 2022). Finally, *Enterococcus*, *Terrisporobacter*, *Escherichia‐Shigella* and *Klebsiella* are all host‐associated bacteria that have already been found in snowpack and cold environments due to animal faecal contamination (Dancer et al. 1997; Lebreton et al. 2014; Staley et al. 2017; Kalinowska et al. 2021; Niu et al. 2023). However, while *Enterococcus* and *Terrisporobacter* are ubiquitous mammalian gut microbiome symbionts (Lebreton et al. 2014; Niu et al. 2023), *Escherichia–Shigella* and *Klebsiella* can acquire opportunistic pathogenic behaviour. These opportunistic pathogens of animal origin have been shown to accumulate at > 1000 CFU/mL in the snowpack from uninhabited and unpressurised sites and remain preserved at cold temperatures, persisting for long periods with potential health implications (Dancer et al. 1997; Lebreton et al. 2014; Staley et al. 2017; Kalinowska et al. 2021; Niu et al. 2023). Taken together, these data confirm the complexity of the snow microbiome ecosystem (Bourquin et al. 2022), where microorganisms with different ecological propensities can coexist, playing different ecological roles. For instance, cold‐adapted microorganisms that actively metabolise nutrients available in the snowpack (Malard et al. 2023) may be important for biogeochemical cycling and circularisation of nutrients in the snow ecosystem. Differently, non‐psychrophilic generalists (Zhu et al. 2020), preserved in the snow environment, may become part of the soil microbiome at melting and play an important role in soil fertilisation. Finally, host‐associated taxa that accumulate in the snowpack could exploit snow as a dispersal system, facilitating inter‐host dispersal processes.

Through LDA, we then identified several ASVs that specifically characterised the impacted site. In particular, the in‐track impacted site was discriminated by ASVs assigned to *Terrisporibacter*, *Clostridium sensu stricto 1*, *Acetobacteraceae*, *Citrobacter*, *Muribaculaceae*, *Methylobacterium–Methylorubrum* and *Enterococcus*. While *Acetobacteraceae* and *Methylobacterium–Methylorubrum* are known N_2_‐fixing microorganisms (Alessa et al. 2021; Carey et al. 2016)—with the former being characteristic of cryospheric environments—the remaining taxa are all host associated, such as the gut symbionts *Terrisporobacter*, *Clostridium sensu stricto 1*, *Enterococcus* and *Muribaculaceae* (Lebreton et al. 2014; Lagkouvardos et al. 2019; Lanthier et al. 2021; Niu et al. 2023) and the opportunistic pathogen *Citrobacter* (Samonis et al. 2009; Collins et al. 2014). It should be noted that *Enterococcus* was detected at a relative abundance of 32.32% at the in‐track impacted site in March, which we consider to be of particular biological relevance. Indeed, considering the prokaryotic abundances in the March samples from this site (8.12 ± 0.49 × 10^4^ cells/mL), we can infer an absolute abundance of *Enterococcus* of about 2.6 × 10^4^ cells/mL, suggesting a relevant accumulation of these bacteria in the snow track at the end of the season. Interestingly, *Enterococcus* has already been observed in artificial snow, derived from the contamination of river water used for artificial snow production, and has been shown to be able to survive the artificial snow production process, then populating the snow ecosystem in the track after snow deposition (Lenart‐Boroń et al. 2017). Our hypothesis is that, at Santa Caterina Valfurva, the large number of tourists during the winter season could lead to the overloading of municipal wastewaters with faecal bacteria, favouring the contamination of nearby rivers. Alternatively, faecal bacteria contamination could originate from farms near the river, which would release microorganisms into the water, contaminating it (Scicchitano, Babbi, et al. 2024; Scicchitano, Leuzzi, et al. 2024). The usage of these river waters for the production of artificial snow could lead to the accumulation of host‐derived microorganisms (e.g., *Enterococcus*) capable of resisting artificial snow production in the snowpack of ski tracks. According to the 2023 Legambiente dossier (https://www.legambiente.it/wp‐content/uploads/2021/11/Report‐Nevediversa_2023.pdf), during the study period, artificial snow production in the Italian Alps consumed approximately 96.84 million cubic metres of water. With around 90% of ski slopes being artificially snow covered, this highlights the extensive and growing reliance on artificial snow in alpine tourism.

Finally, through inferred metagenomics, we found that the taxa characterising the impacted site, both inside and outside the ski track, were endowed with genes for the degradation of several xenobiotic compounds, including benzoate, bisphenol, styrene and caprolactam, which are common anthropic contaminants of ski tracks. Indeed, benzoate moieties are present in polyesters, which are the most widely used material for apparel textiles. Caprolactam is the monomer used in the production of Nylon 6, one of the most widely used materials for the production of fibres and ski‐bindings, while styrene is present in ABS polymers, which are used to make ski side walls. Finally, the benzoate and bisphenol degradation pathways may be associated with the breakdown of aromatic additives found in modern ski waxes.

In conclusion, our study provided some glimpses on the complexity and multifunctionality of the snow microbiome, where microorganisms with different ecological propensities can coexist, providing functions with ecosystem roles that extend far beyond the Alpine cryosphere. Furthermore, we suggest that winter tourism has a significant impact on the snow ecosystem, promoting the selection and enrichment of host‐associated and xenobiotic‐degrading microorganisms. We hypothesise that ski tourism introduces multiple, interacting anthropogenic stressors that reshape the snow microenvironment. These include the widespread use of artificial snow, characterised by distinct chemical and microbiological profiles compared to natural snow and potentially introducing allochthonous bacteria and viruses, as well as the physical activity of skiing itself, which leads to the release of various xenobiotic compounds originating from sports equipment and ski wax, contributing to the enrichment of specific xenobiotic‐degrading taxa. Our data highlight the need for systematic monitoring of the impact of winter tourism on snow‐associated microbial communities. This activity is particularly urgent in the context of growing overtourism, such as in the Italian Alps, and the increasingly intensive use of artificial snowmaking. It is essential to extend monitoring efforts to all alpine biomes, using an integrated approach that considers the snow microbiota as a key indicator, in order to assess systemic impacts on environmental, animal, and human health, fully aligned with the One Health framework.

The adoption of sustainable practices, both in terms of materials used for alpine skiing and snowmaking technologies, should be evaluated with the goal of minimising the environmental impact of winter tourism on alpine ecosystems and their associated microbiota. Where ecosystems are already compromised, targeted environmental restoration measures should be implemented to recover ecosystem health.

## Author Contributions

**Andrea Nicolò Dell'Acqua:** data curation, formal analysis, visualization, writing – original draft. **Daniel Scicchitano:** data curation, formal analysis, visualization, writing – review and editing. **Nicola Simoncini:** data curation, formal analysis, writing – review and editing. **Ilaria Mercanti:** writing – review and editing. **Daniela Leuzzi:** writing – review and editing. **Silvia Turroni:** writing – review and editing. **Luca Corlatti:** conceptualization, data curation, formal analysis, project administration, supervision, resources, writing – review and editing. **Simone Rampelli:** writing – review and editing. **Martino Colonna:** conceptualization, data curation, formal analysis, project administration, supervision, resources, writing – review and editing. **Cinzia Corinaldesi:** conceptualization, project administration, supervision, resources, writing – review and editing. **Marco Candela:** conceptualization, project administration, supervision, resources, writing – original draft. **Giorgia Palladino:** data curation, formal analysis, visualization, writing – original draft.

## Conflicts of Interest

The authors declare no conflicts of interest.

## Supporting information


**Figure S1:** Meteorological conditions at the sampling sites during the sampling period (December 2021–April 2022). On top, daily snow depth and minimum air temperature at a sampling site near the Santa Caterina ski area (46°26′22″, 10°23′23″). As the Santa Caterina ski area did not have a meteorological station, we show the data collected by a meteorological station in a neighbouring ski area (Bormio 2000), with similar characteristics in terms of elevation (2000 m a.s.l.) and exposure (north). On bottom, daily snow depth and minimum air temperature at the Cancano reference sampling site.

**Figure S2:** Epifluorescence microscopy image of a snow sample. Samples were filtered onto a Whatman 0.02 μm Anodisc filter and stained with SYBR Gold, showing viruses (0.02–0.2 μm) and prokaryotic cells (0.2–2 μm).

**Figure S3:** Compositional structure of the snow‐associated microbiome in the different sampling sites. Principal coordinates analysis (PCoA) based on the Bray–Curtis distances at the family level between microbial profiles of snow collected at the reference site (‘Ref’) and at the two impacted sites (‘In’ and ‘Out’ of the ski track) in the different sampling months (from December 2021 to April 2022). The first and second principal components (PCo1 and PCo2) are plotted and the percentage of variance in the dataset explained by each axis is shown. Permutation test with pseudo‐*F* ratio, *p* > 0.05 for all comparisons.

**Figure S4:** Metabolic potential of the discriminating ASVs of the impacted sites. Complete linkage method based on the Euclidean correlation of the KO (KEGG Orthology) genes belonging to KEGG pathways assigned to ‘Infectious diseases; bacterial’ and ‘Xenobiotic biodegradation and metabolism’, predicted for the ASVs characteristic of the in‐track (red) and out‐track (orange) impacted sites. Discriminating ASVs were retrieved by linear discriminant analysis (LDA) effect size (LEfSE, Figure 5). The relative *Z*‐score is reported. Single KOs and correspondent functions are reported in the table on the right.

**Table S1:** Statistical analysis of prokaryotic and viral abundances at the sampling sites in January and March 2022. Analysis of variance (ANOVA) was used to investigate differences in prokaryotic and viral abundances among stations (factor type: reference site ‘Ref’, impacted site in‐track ‘Impact_in’, impacted site out‐track ‘Impact_out’) and between sampling months (factor month: January ‘Jan’ and March ‘Mar’). Whenever factors were identified as significant, a HSD‐Tukey post hoc test was performed.

**Table S2:** Shared ASVs between sampling sites and timepoints. ID and corresponding taxonomy of ASVs clustered at 97% similarity shared between different timepoints at the reference site, the in‐track impacted site, and the out‐track impacted site, and those shared among all sampling sites encompasing the enritre sampling period (from December 2021 to April 2022; overall shared). See also Figure 4.

## Data Availability

High‐quality reads from the samples sequenced in this study were deposited in the European Nucleotide Archive under the project accession number ENA: PRJEB79922.
