# Supplementary material for: Ski Tourism Shapes the Snow Microbiome on Ski Slopes in the Italian Central Alps
Source: Environ Microbiol Rep. 2025 Sep 18;17(5):e70195. doi: 10.1111/1758-2229.70195 (PMC12444944; doi:10.1111/1758-2229.70195)
Supplement: Supplementary file 1 — Figure S1: Meteorological conditions at the sampling sites during the sampling period (December 2021–April 2022). On top, daily snow depth and minimum air temperature at a sampling site near the Santa Caterina ski area (46°26′22″, 10°23′23″). As the Santa Caterina ski area did not have a meteorological station, we show the data collected by a meteorological station in a neighbouring ski area (Bormio 2000), with similar characteristics in terms of elevation (2000 m a.s.l.) and exposure (north). On bottom, daily snow depth and minimum air temperature at the Cancano reference sampling site. [file EMI4-17-e70195-s001.pdf]

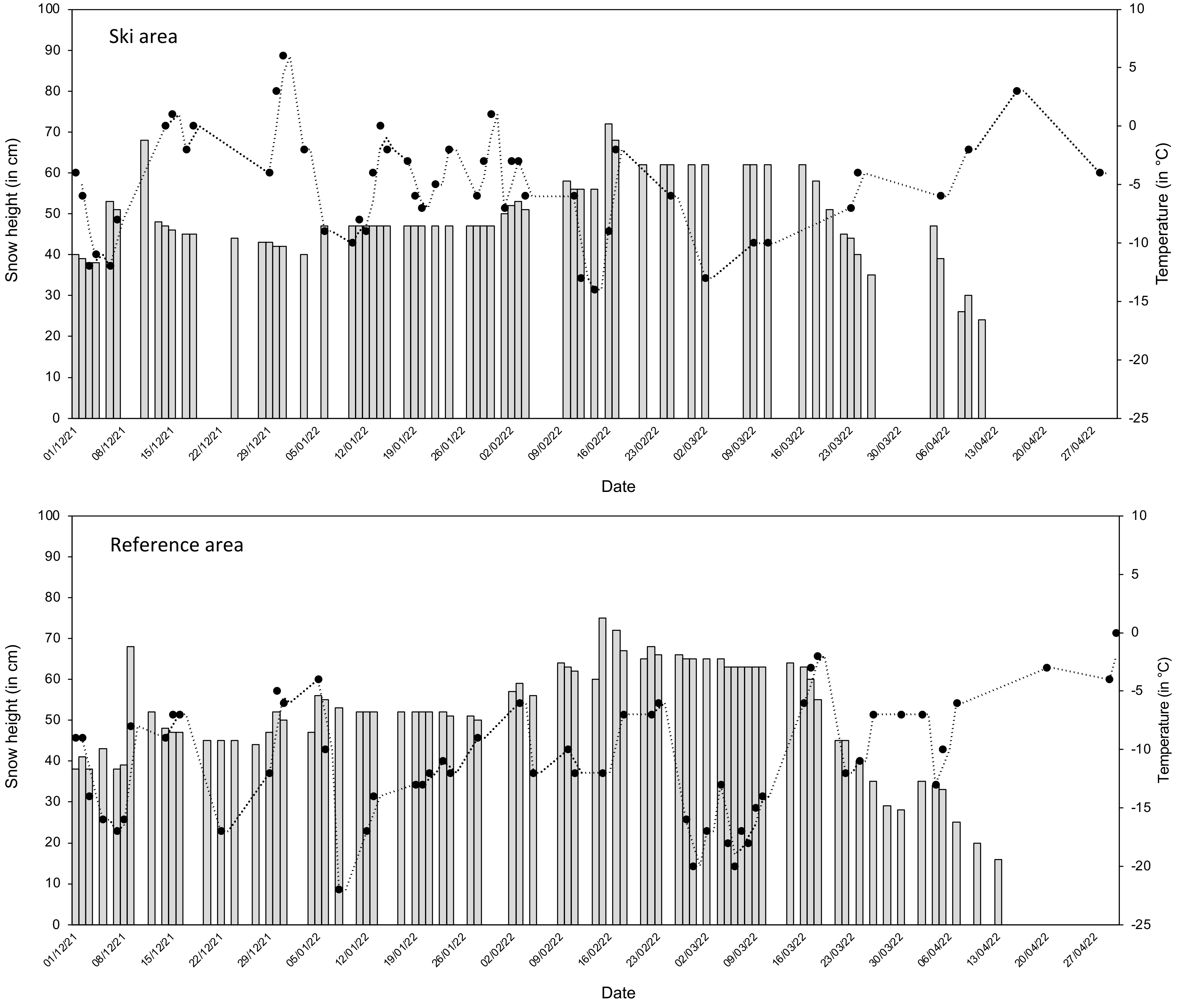


**Supplementary Figure 1 - Meteorological conditions at the sampling sites during the sampling period (December 2021 - April 2022).** On top, daily snow depth and minimum air temperature at a sampling site near the Santa Caterina ski area (46°26'22", 10°23'23"). As the Santa Caterina ski area did not have a meteorological station, we show the data collected by a meteorological station in a neighboring ski area (Bormio 2000), with similar characteristics in terms of elevation (2000 m a.s.l.) and exposure (north). On bottom, daily snow depth and minimum air temperature at the Cancano reference sampling site.
