# Supplementary material for: Ski Tourism Shapes the Snow Microbiome on Ski Slopes in the Italian Central Alps
Source: Environ Microbiol Rep. 2025 Sep 18;17(5):e70195. doi: 10.1111/1758-2229.70195 (PMC12444944; doi:10.1111/1758-2229.70195)
Supplement: Supplementary file 2 — Figure S2: Epifluorescence microscopy image of a snow sample. Samples were filtered onto a Whatman 0.02 μm Anodisc filter and stained with SYBR Gold, showing viruses (0.02–0.2 μm) and prokaryotic cells (0.2–2 μm). [file EMI4-17-e70195-s004.pdf]

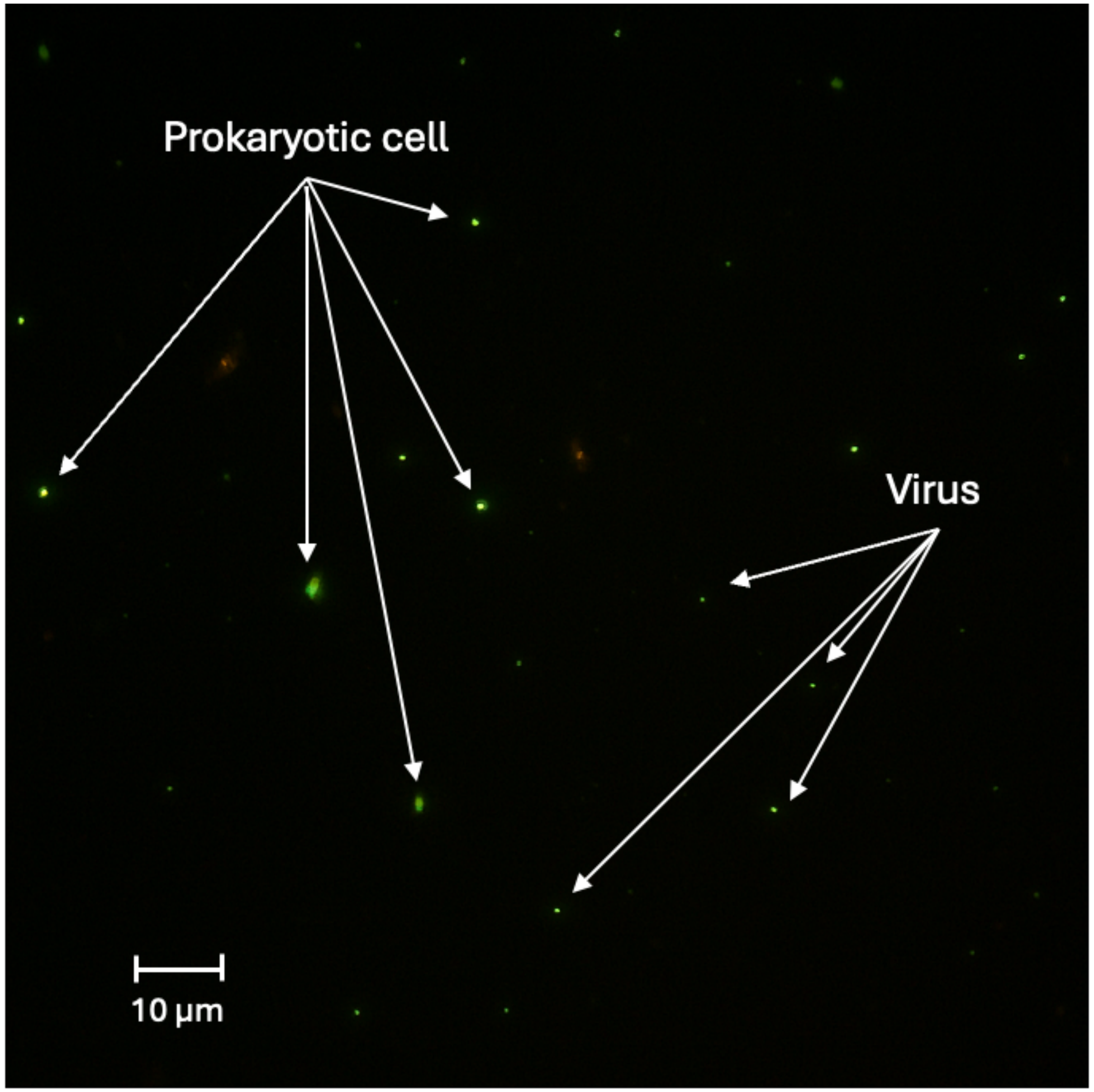

Supplementary Figure 2 - Epifluorescence microscopy image of a snow sample. Samples were filtered onto a Whatman 0.02 µm Anodisc filter and stained with SYBR Gold, showing viruses (0.02–0.2 µm) and prokaryotic cells (0.2–2 µm).
