# Supplementary material for: Ski Tourism Shapes the Snow Microbiome on Ski Slopes in the Italian Central Alps
Source: Environ Microbiol Rep. 2025 Sep 18;17(5):e70195. doi: 10.1111/1758-2229.70195 (PMC12444944; doi:10.1111/1758-2229.70195)
Supplement: Supplementary file 3 — Figure S3: Compositional structure of the snow‐associated microbiome in the different sampling sites. Principal coordinates analysis (PCoA) based on the Bray–Curtis distances at the family level between microbial profiles of snow collected at the reference site (‘Ref’) and at the two impacted sites (‘In’ and ‘Out’ of the ski track) in the different sampling months (from December 2021 to April 2022). The first and second principal components (PCo1 and PCo2) are plotted and the percentage of variance in the dataset explained by each axis is shown. Permutation test with pseudo‐F ratio, p > 0.05 for all comparisons. [file EMI4-17-e70195-s003.pdf]

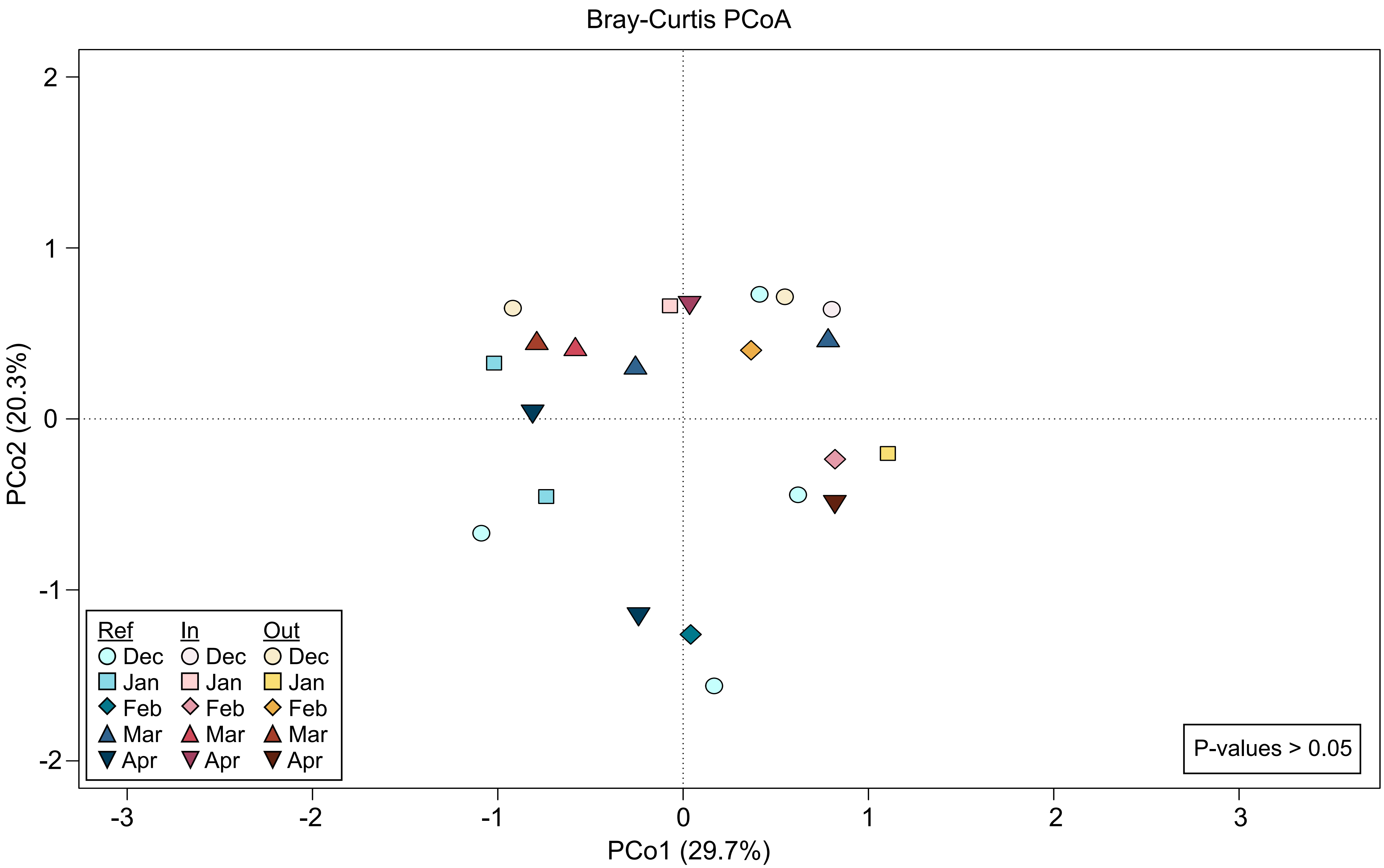

Supplementary Figure 3 - Compositional structure of the snow-associated microbiome in the different sampling sites. Principal coordinates analysis (PCoA) based on the Bray-Curtis distances at the family level between microbial profiles of snow collected at the reference site ("Ref") and at the two impacted sites ("In" and "Out" of the ski track) in the different sampling months (from December 2021 to April 2022). The first and second principal components (PCo1 and PCo2) are plotted and the percentage of variance in the dataset explained by each axis is shown. Permutation test with pseudo-F ratio, p-value > 0.05 for all comparisons.
