# Supplementary material for: Ski Tourism Shapes the Snow Microbiome on Ski Slopes in the Italian Central Alps
Source: Environ Microbiol Rep. 2025 Sep 18;17(5):e70195. doi: 10.1111/1758-2229.70195 (PMC12444944; doi:10.1111/1758-2229.70195)
Supplement: Supplementary file 4 — Figure S4: Metabolic potential of the discriminating ASVs of the impacted sites. Complete linkage method based on the Euclidean correlation of the KO (KEGG Orthology) genes belonging to KEGG pathways assigned to ‘Infectious diseases; bacterial’ and ‘Xenobiotic biodegradation and metabolism’, predicted for the ASVs characteristic of the in‐track (red) and out‐track (orange) impacted sites. Discriminating ASVs were retrieved by linear discriminant analysis (LDA) effect size (LEfSE, Figure 5). The relative Z‐score is reported. Single KOs and correspondent functions are reported in the table on the right. [file EMI4-17-e70195-s005.pdf]

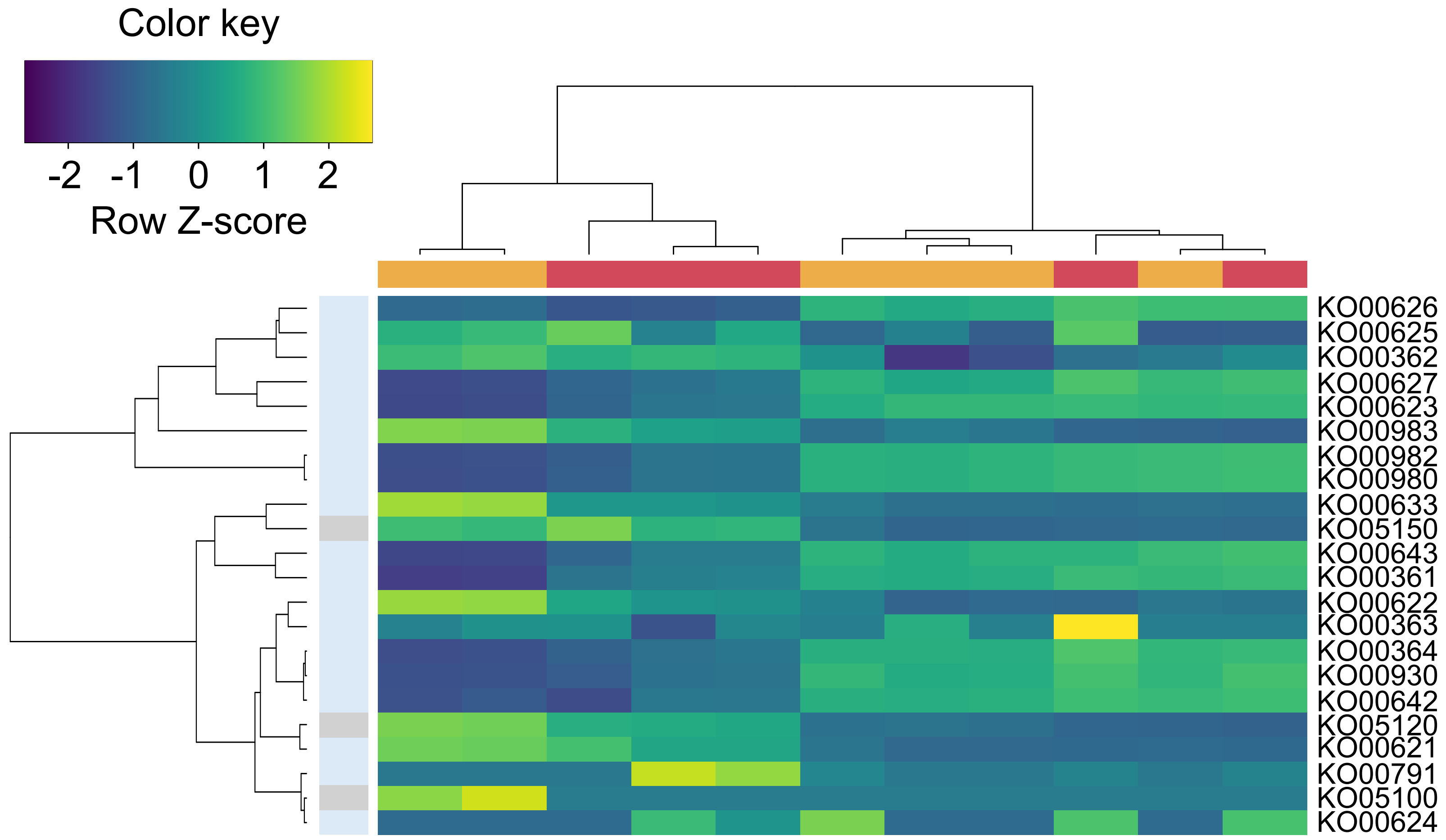

KO	KEGG Class	Function
ko05120	Infectious disease: bacterial	Epithelial cell signaling in Helicobacter pylori infection
ko05150		Staphylococcus aureus infection
ko05100		Bacterial invasion of epithelial cells
ko00625	Xenobiotics biodegradation and metabolism	Chloroalkane and chloroalkene degradation
ko00624		Polycyclic aromatic hydrocarbon degradation
ko00627		Aminobenzoate degradation
ko00626		Naphthalene degradation
ko00621		Dioxin degradation
ko00623		Toluene degradation
ko00622		Xylene degradation
ko00633		Nitrotoluene degradation
ko00363		Bisphenol degradation
ko00364		Fluorobenzoate degradation
ko00361		Chlorocyclohexane and chlorobenzene degradation
ko00362		Benzoate degradation
ko00643		Styrene degradation
ko00642		Ethylbenzene degradation
ko00980		Metabolism of xenobiotics by cytochrome P450
ko00983		Drug metabolism - other enzymes
ko00791		Atrazine degradation
ko00982		Drug metabolism - cytochrome P450
ko00930		Caprolactam degradation

Supplementary Figure 4 - Metabolic potential of the discriminating ASVs of the impacted sites. Complete linkage method based on the Euclidean correlation of the KO (KEGG Orthology) genes belonging to KEGG pathways assigned to "Infectious diseases; bacterial" and "Xenobiotic biodegradation and metabolism", predicted for the ASVs characteristic of the in-track (red) and out-track (orange) impacted sites. Discriminating ASVs were retrieved by linear discriminant analysis (LDA) effect size (LEfSE, Figure 5). The relative *Z*-score is reported. Single KOs and correspondent functions are reported in the table on the right.
